# Blood-based liquid biopsy: insights into early detection, prediction, and treatment monitoring of bladder cancer

**DOI:** 10.1186/s11658-023-00442-z

**Published:** 2023-04-04

**Authors:** Shijie Li, Kerong Xin, Shen Pan, Yang Wang, Jianyi Zheng, Zeyu Li, Xuefeng Liu, Bitian Liu, Zhenqun Xu, Xiaonan Chen

**Affiliations:** 1 Department of Urology, Shengjing Hospital of China Medical University, Shenyang, Liaoning 110004 People’s Republic of China; 2 Department of Radiology, Shengjing Hospital of China Medical University, Shenyang, Liaoning 110004 People’s Republic of China; 3 Department of Gynecology, Cancer Hospital of China Medical University, Liaoning Cancer Hospital & Institute, Shenyang, 110042 Liaoning People’s Republic of China

**Keywords:** Bladder cancer, Liquid biopsy, Circulating tumor cells, Circulating tumor DNA, Cell-free RNA, Exosomes, Metabolomics, Proteomics, Clinical application

## Abstract

Bladder cancer (BC) is a clinical challenge worldwide with late clinical presentation, poor prognosis, and low survival rates. Traditional cystoscopy and tissue biopsy are routine methods for the diagnosis, prognosis, and monitoring of BC. However, due to the heterogeneity and limitations of tumors, such as aggressiveness, high cost, and limited applicability of longitudinal surveillance, the identification of tumor markers has attracted significant attention in BC. Over the past decade, liquid biopsies (e.g., blood) have proven to be highly efficient methods for the discovery of BC biomarkers. This noninvasive sampling method is used to analyze unique tumor components released into the peripheral circulation and allows serial sampling and longitudinal monitoring of tumor progression. Several liquid biopsy biomarkers are being extensively studied and have shown promising results in clinical applications of BC, including early detection, detection of microscopic residual disease, prediction of recurrence, and response to therapy. Therefore, in this review, we aim to provide an update on various novel blood-based liquid biopsy markers and review the advantages and current limitations of liquid biopsy in BC therapy. The role of blood-based circulating tumor cells, circulating tumor DNA, cell-free RNA, exosomes, metabolomics, and proteomics in diagnosis, prognosis, and treatment monitoring, and their applicability to the personalized management of BC, are highlighted.

## Introduction

Bladder cancer (BC) poses a huge social burden, with a global yearly occurrence of more than 430,000 cases, resulting in nearly 170,000 deaths annually [1]. It is a highly heterogeneous malignancy, especially in advanced stages [2]. BC can present as non-muscle-invasive bladder cancer (NMIBC), muscle-invasive bladder cancer (MIBC), or metastatic disease incidents, each with different molecular drivers. NMIBC accounts for approximately 75% of all BC types, and at least half of patients with NMIBC have a high rate of recurrence and disease progression to MIBC within 5 years [3]. Additionally, MIBC has a high propensity to spread to lymph nodes and other organs, and approximately half of patients with MIBC develop metastases and die within 3 years [4, 5]. The high morbidity and mortality of BC is in part associated with the lack of effective early diagnosis and prognostic approaches. Therefore, early diagnosis and lifelong surveillance are clinically important to improve the long-term survival of patients with BC.

Currently, invasive cystoscopy and tissue biopsy remain the gold standard for BC identification and surveillance. However, the drawbacks of this method, such as sampling bias, invasiveness, and difficulty in sampling deep tumors, limit its use in large-scale screening. Additionally, urine cytology represents another diagnostic possibility but is limited by poor sensitivity, especially for low-grade tumors [6, 7]. Other imaging tests are limited to BC staging in clinical practice owing to increased ionizing radiation and/or cost, and there is often a delay in identifying recurrence and metastasis with imaging [8]. Therefore, there is an urgent need for biomarkers with high specificity and sensitivity that can be applied to BC.

**Fig. 1:**
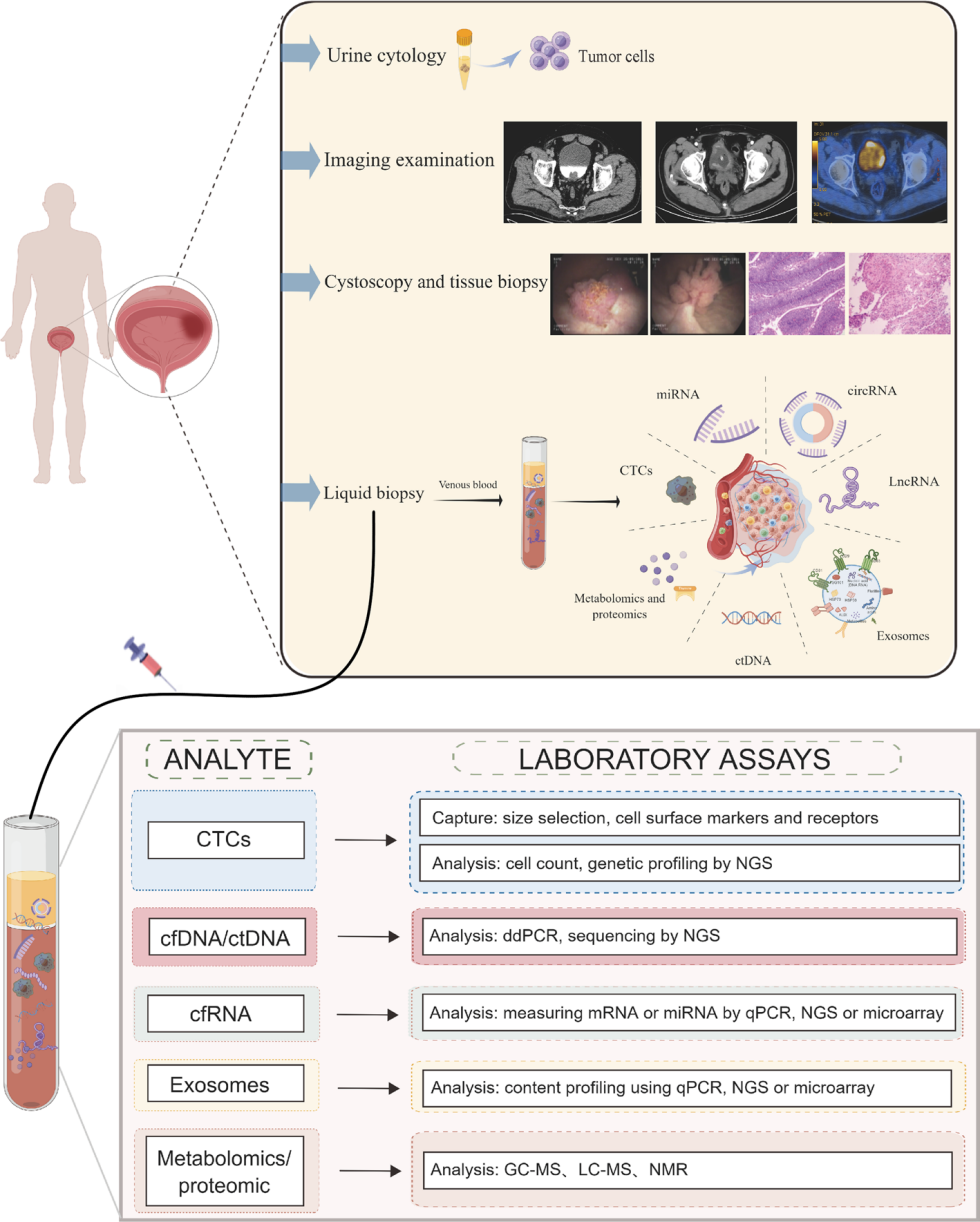
Overview of clinical examination methods for bladder cancer and laboratory analytical techniques for liquid biopsies. Routine imaging tests used for bladder cancer detection and diagnosis include X-ray, CT, and PET–CT. Cystoscopy and tissue biopsy are the gold standard for the diagnosis of bladder cancer. Urine cytology is available as a complementary test. Liquid biopsy is emerging as a promising method. Liquid biopsy involves the collection and analysis of five different tumor components from peripheral blood samples: CTCs, cell-free nucleic acids (cfDNA/ctDNA, cfRNA), exosomes, and metabolomics and proteomics. Tumor components are then captured and analyzed in peripheral blood samples using appropriate laboratory assays. *CT* computed tomography, *PET* positron emission computed tomography, *CTCs* circulating tumor cells, *ctDNA* circulating tumor deoxyribonucleic acid, *cfDNA* cell-free DNA, *cfRNA* cell-free RNA

Recently, genomic profiles of blood and body fluids based on liquid biopsies have been shown to be highly correlated with the genomic profiles of tumors [9–11]. DNA fragments containing tumor-specific alterations, including information about point mutations, DNA methylation, and copy number variation, are released into circulation by tumors. The dynamic assessment of specific molecular markers by liquid biopsy allows for real-time monitoring of tumors, thus facilitating the selection of targeted therapies. In contrast to cystoscopy and tissue biopsy, liquid biopsy is a holistic examination of blood and body fluids, reflecting the overall expression of tumor cells [12]. Figure 1 summarizes the current testing methods applied to BC and the laboratory analytical techniques of liquid biopsy [13, 14].

Liquid biopsy is based on the detection of circulating tumor cells (CTCs) and “cell-free” nucleic acids in blood and body fluids, including urine, saliva, tears, sweat, amniotic fluid, cerebrospinal and pleural fluids, and cervicovaginal secretions, to detect and quantify targets of interest [13, 14]. Significant progress has been made in the detection of urinary biomarkers for BC, but it is currently limited by its low sensitivity and specificity [15, 16]. Since blood is in contact with most tumors, liquid biopsies mainly involve blood sampling. Liquid biopsies contribute to better characterization of BC by identifying tumor cells or tumor DNA that is released into the bloodstream with tumor progression or regression [17]. Meanwhile, unlike urine, the blood-based biomarkers or liquid biopsies can be applied to all BC, but may be particularly useful after cystectomy for curative purposes, providing a strategy for detecting minimal residual disease (MRD) prior to conventional imaging tests. The highly sensitive technique of blood testing provides detail of tumor characteristics, and blood-based cancer genomics may not be limited by sampling frequency, tumor availability, or the presence of a clinically significant disease compared with tissue sampling.

Historically, it is difficult to determine potentially recurrent or successfully cured patients with BC after surgery. This limitation has resulted in the unnecessary exposure of successfully cured patients to the toxicity of adjuvant therapy, whereas others with residual disease may not receive potentially beneficial treatment until disease progression can be detected by imaging. A comprehensive understanding of the subgroup of patients predicted to become resistant to cisplatin is essential for patient treatment modalities [18]. The advent of immunotherapy, especially immune checkpoint inhibitors (ICIs), has revolutionized the landscape of BC systemic therapy over the past 5 years. However, ICIs provide durable immune memory in only a minority of patients [19]; additionally, most patients exhibit poor or no response to ICIs, and those who initially respond eventually develop resistance and disease progression. Moreover, ICIs are expensive and impose a significant financial burden on patients. The occurrence of immune-related adverse effects can be fatal and may lead to permanent disease [20]. Therefore, there is a need to use predictive biomarkers of response to ICIs to assess early efficacy and toxicity of drugs and to better understand resistance mechanisms to guide therapeutic decisions. Studies have shown that liquid biopsies can be applied to comprehensively examine tumor genome and monitor genetic variation dynamically during treatment [21–23]. Overall, liquid biopsy can help in the selection of patients for immunotherapy.

**Fig. 2:**
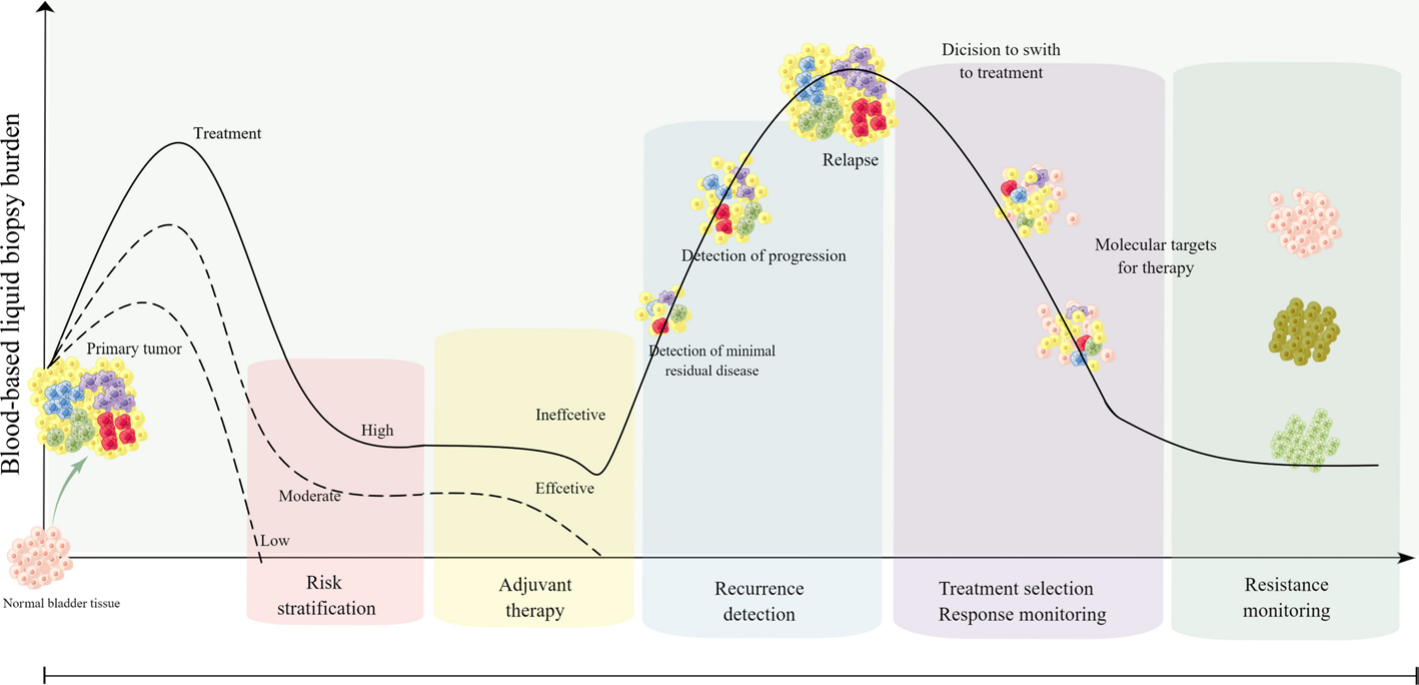
Overview of the clinical use of liquid biopsy in bladder cancer. Sample analysis of bladder cancer biomarkers early in the disease process allows for early diagnosis and risk stratification. After receiving surgery, liquid biopsy can detect minimal residual disease as a prognostic indicator and allow early detection of recurrent disease. During treatment, liquid biopsy can enhance longitudinal surveillance through its noninvasive approach, thus enabling continuous sampling. In addition, liquid biopsies have the advantage of capturing the entire tumor genome, which can help identify novel genetic markers for targeted therapy and detect treatment resistance

Currently, liquid biopsies usually detect CTCs, exosomes, circulating tumor deoxyribonucleic acid (ctDNA), microRNAs (miRNAs), long non-coding RNAs (lncRNAs), circular RNAs (circRNAs), extracellular vesicles (EVs), tumor-educated platelets, proteins, and metabolites [23–25]. New evidence for the clinical utility of liquid biopsies from noninvasive blood tests in BC continues to emerge and may provide a genomic profile that can be utilized in patients with BC and guide their treatment in some ways (Fig. 2). The purpose of this review is to discuss the latest information on liquid biopsy components and their clinical utility for early diagnosis of BC, prognosis, monitoring of cancer treatment outcomes, detection of early recurrence, and individualized treatment selection.

### CTC

The concept of CTCs was first described by the Australian scholar Ashworth in 1869, and it was defined as tumor cells shed from primary tumors or metastatic lesions and cleared by the lymphatic or circulatory system [26]. To date, most studies on CTCs have focused on the exploration of CTCs in the blood circulation, which are few in number (up to a few hundred per milliliter), depending on the available detection/isolation techniques and the definition of CTCs used.

After release from the primary tumor, the process by which CTCs survive in the circulatory system and spread to remote organs requires overcoming several obstacles, with less than 0.01% of CTCs released into the circulation eventually surviving and producing metastases [27]. First, solid tumors shed tumor cells that normally cross the endothelium into the circulation via epithelial–mesenchymal transition (EMT), a highly dynamic process of epithelial to quasi-mesenchymal cell transformation with enhanced metastatic potential and invasive capacity and resistance to some therapeutic strategies [28, 29]. Thereafter, most CTCs experience necrosis or apoptosis owing to environmental challenges in the blood, such as hemodynamic shear or immune system attack, with only a small percentage of CTCs interacting closely with cancer-associated fibroblasts, neutrophils or platelets, eventually evading the immune system and surviving [30–32]. Lastly, CTCs must evade the defenses of the natural immune system and recognition by natural killer cells [33, 34].

Since most cancers are epithelial in nature, the most commonly used marker for CTCs is epithelial cell adhesion molecule (EpCAM), a “universal” marker of cancer [35]. Several studies have shown the predictive value of EpCAM-based CTC assays in breast cancer and non-small cell lung cancer, which are cancers that strongly express EpCAM [36, 37]. Moreover, high-grade advanced stages of BC were significantly associated with EpCAM expression, and EpCAM expression was associated with poor overall survival. The strong association of EpCAM expression with high-grade tumors suggests a possible role in tumor progression, making EpCAM a potential target for antibody-mediated therapy [38]. Additionally, EpCAM was found to be a viable imaging target for the diagnosis of lymph node metastases in migratory cell carcinoma of the bladder. Perioperative examination of these metastases could improve disease staging and increase the rate of complete resection of MIBC lymph node metastases. Overall, these findings indicate that EpCAM-positive CTCs could be a robust and sensitive biomarker [39].

CTCs detected via liquid biopsy contain the complete genomic and transcriptomic information of cancer cells, making CTCs suitable clinical biomarkers [40]. The application of CTC in tumors has many advantages, including the analysis of genetic information or tumor morphology, which can improve patient classification and stratification, effective monitoring of patient response, and improved clinical prognosis [33, 41]. Additionally, CTCs can be selectively recovered from patients’ blood, amplified in vitro, and used to generate relevant animal models, enabling drug sensitivity assessment, biomarker discovery, and personalization of targeted therapies. These features suggest that blood detection of CTCs may be important for the clinical management of BC, facilitating the identification of tumor heterogeneity, potential micrometastases, and tumor evolution over time [42–44].

Over the last two decades, emerging isolation techniques have enabled the biological study of CTCs and promote their use in clinical applications for screening, treatment response monitoring, and prognostic assessment of cancer. Techniques for isolating CTCs include both physical property-based and biologic property-based approaches. The physical separation of CTCs for enrichment is based on the differences between CTCs and blood cells in size (filter-based devices), density, deformability, and charge (electrophoresis) [45]. Biological property-based techniques include immunoaffinity methods that rely on antibody–antigen interactions, in which CTCs are enriched positively by EpCAM and interstitial material (vimentin) or negatively by the leukocyte-specific antigen CD45 to remove unwanted leukocytes [46]. The CellSearch system is the most frequently used technique for the detection of CTCs and it is an immunoaffinity-based isolation strategy for identifying CTCs on the basis of EpCAM-positive expression [47]. However, a previous study found that only about 32% of patients with BC were EpCAM positive [48]. Moreover, CTCs could not be detected in the blood of patients with nonmetastatic BC [49]. Therefore, the application of CellSearch in BC may be limited in the case of patients with low expression of CTCs. However, some novel techniques, such as nanotechnology-based techniques and microfluidic chips, have been developed and are expected to be examined in clinical studies [50, 51]. Despite the advantages and disadvantages between the different methods, the effective integration of such technologies may facilitate CTC research in several aspects, particularly in terms of in-depth analyses and potential clinical utilization. Although the application of CTC assays for BC still has technical challenges, they are being evaluated in clinical trials as tools for predictive and response biomarkers and MRD surveillance.

### CTCs in BC diagnosis/prognosis

CTCs are released into the circulation during the early cancer stages; therefore, the detection of CTCs could have considerable clinical application for the early diagnosis of several types of BC. One study detected CTCs by tumor-specific ligand PCR and evaluated their utility in the diagnosis of BC. The sensitivity and specificity of CTC quantitative assay for BC based on high expression of folate receptor-α were 82.1% and 61.9, with an area under the receiver operating characteristic curve (AUC) of 0.819 [52]. In another study, after next-generation sequencing (NGS) of matched BCs and CTCs, *KMT2C* mutations were detected among those commonly observed in CTCs and corresponding primary tumors [53]. In contrast, the *KMT2C* gene is frequently observed to be mutated in patients with MIBC [54], indicating the potential clinical value of CTCs in the early identification of high-risk BC.

Several studies have evidenced that the presence of CTCs is associated with increased disease recurrence, post-radical cystectomy (RC) cancer-specific survival (CSS) and overall survival (OS). The detection of CTCs is a strong predictor of disease progression and poor prognosis in early BC [55–61]. A recent metaanalysis of 30 studies showed that the presence of CTCs in peripheral blood is an independent predictor of poor prognosis in patients with BC [62]. Similarly, the presence of CTCs has been shown to be an independent predictor of disease progression to muscle-invasive disease in a large and homogeneous group of patients with high-risk NMIBC, with a 75% predictive value [56]. Lin et al. used the CellSearch system to analyze CTCs in 188 patients with BC, and found that approximately one-quarter had detectable CTC prior to RC, and CTC-positive patients treated with RC (with or without adjuvant chemotherapy) had poorer progression free survival (PFS), CSS, and OS compared with CTC-negative patients [56].

It was reported that preoperative detection of any CTCs predicts a higher risk of recurrence and poorer CSS in RC-treated patients with clinically non-metastatic BC compared with CTC-negative patients [58]. Similarly, in a recent study, Beije et al. sought to explore the predictive value of CTCs in the prognostic value of patients with non-metastatic MIBC after RC. They found a significant decrease in OS, CSS, and recurrence-free survival (RFS) in patients with positive CTCs [63]. In addition, Soave et al. investigated the potential role of CTC status on adjuvant chemotherapy (AC) management decisions in patients with BC after RC, and found that the presence of CTC was associated with poorer PFS, CSS, and OS in patients not on AC, indicating that CTCs may be useful in decisions for or against AC [61]. Gazzaniga et al. found that CTCs were detectable in approximately 18% of NMIBC, and that CTC levels could be used to distinguish patients at high risk of recurrence from those at high risk for progression, facilitating the early identification of candidate patients for adjuvant therapy after RC [59]. Additionally, Osman et al. found that patients with urea/*EGFR*-positive CTCs after RC had a higher risk of recurrence [64].

Several studies have detected CTCs in high-risk or progressive BC, and have reported a correlation between CTCs and tumor stage, lymph node metastasis, and survival [42, 65–68]. CTCs were identified in 19% of patients with advanced BC undergoing RC (with or without perioperative chemotherapy), and the presence of CTCs was associated with increased risk of disease progression; moreover, imaging metastatic disease was found to be significantly correlated with the presence of CTCs [65]. Additionally, higher levels of CTCs were detected in MIBC compared with NMIBC, suggesting that these cells may predominate during tumor muscle infiltration and disease progression [66]. Gradilone et al. found that CTCs could be isolated in the blood of 24 of 54 (44%) patients with T1G3 BC, and that CTC positivity was an independent prognostic factor (*p* < 0.001) of disease-free survival (DFS) [67]. Similarly, Nicolazzo et al. concluded that the presence of CTCs significantly affected disease-free survival and tumor-specific survival (*p* < 0.0001) on the basis of the findings of a long-term follow-up study of patients with T1G3 BC [68].

The identification of CTCs is considered a promising complementary tool for risk stratification of BC recurrence and progression, which is essential for the development of optimal surveillance strategies for patients after diagnosis [69–71]. Busetto et al. evaluated 155 patients with BC with pathologically confirmed T1G3 with preoperative CTCs, and observed a strong correlation between the presence of CTCs and time to first recurrence and time to progression, which could be used as a prognostic marker for risk stratification in patients with NMIBC [69]. Nicolazzo et al. demonstrated that the presence of even a single CTC in the peripheral blood of patients with BC indicates a high risk of local recurrence and/or disease progression in patients with ultra-high risk NMIBC [70]. Additionally, CTC counts were found to be significantly higher in the MIBC group than in the NMIBC group, and mortality was significantly higher in patients with high-grade BC than in patients with low-grade BC [71]. These findings indicate that CTC can accurately predict high-risk subgroups of patients, thus allowing longitudinal long-term monitoring of BC.

### CTCs as predictor of treatment response in BC

CTCs may be sensitive biomarkers in assessing chemotherapy response, which could improve the timing of RC in clinical care or changing chemotherapy regimens [58, 63, 72]. Winters et al. conducted a prospective trial using the CellSearch system to examine CTCs in patients with BC treated with cisplatin chemotherapy, and observed a decrease in CTC counts after chemotherapy [72]. Moreover, the absence of CTC in patients with MIBC has been associated with reduced risk of recurrence after cystectomy, as well as tumor-specific death; however, CTC-positive patients with MIBC may benefit more from neoadjuvant chemotherapy [63].

Furthermore, the *HER2* status of CTCs was not consistent with the *HER2* status of the corresponding BC, suggesting that a subgroup of patients with BC with *HER2*-positive CTCs may benefit from *HER2*-targeted therapy. The presence of *HER2*-negative CTCs in patients with *HER2*-positive primary tumors may account for the failure of *HER2*-targeted therapy in a significant number of patients [58].

A recent study showed that baseline and serial assessment of programmed death-ligand 1 (PD-L1) using CTC monitoring may be an effective tool to guide treatment selection in candidate patients for PD-L1 inhibitors after Bacillus Calmette–Guérin (BCG) failure [73]. Serial CTC assays of whole blood could be used to assess the efficacy of checkpoint inhibitor immunotherapies, which are active in a certain percentage of CTC-positive patients and usually have durable responses [74]. Studies regarding blood-based CTCs as potential biomarkers for BC were summarized in Table 1.

**Table 1:** Studies regarding blood-based CTCs as potential biomarkers for bladder cancer

Authors (year)	Sample type	No. of patients	Laboratory technique	Clinical application	Detection rate	Refs.
Qi et al. (2014)	Serum	120	ELISA, RT-qPCR	Diagnostic biomarker	AUC = 81.9%, sensitivity = 82.1%, specificity = 61.9%	[52]
Kim et al. (2020)	Peripheral blood	20	Whole exome sequencing	Discrimination of MIBC from NMIBC and healthy individuals	KMT2C mutations were detected in 20% of CTCs	[53]
Gazzaniga et al. (2014)	Peripheral blood	102	CellSearch System	Predict prognosis	DFS (*p* = 0.005), PFS (*p* = 0.004)	[56]
Soave et al. (2017)	Peripheral blood	188	CellSearch System	Predict prognosis	RFS (*p* < 0.001), CSS (*p* < 0.001)	[57]
Rink et al. (2012)	Peripheral blood	100	CellSearch System	Predict prognosis	OS (*p* = 0.003), CSS (*p* = 0.002), RFS (*p* < 0.001)	[58]
Gazzaniga et al. (2012)	Peripheral blood	44	CellSearch System	Predict prognosis	TFR (*p* < 0.001)	[59]
Rink et al. (2011)	Peripheral blood	50	CellSearch System	Predict prognosis	OS (*p* = 0.001), CSS (*p* < 0.001), PFS (*p* < 0.001)	[60]
Soave et al. (2017)	Peripheral blood	226	CellSearch System	Predict prognosis	OS (*p* < 0.001), CSS (*p* < 0.001), RFS (*p* < 0.001)	[61]
Beije et al. (2022)	Peripheral blood	273	CellSearch System	Predict treatment response	CTC-positive patients treated with NAC have longer survival times	[63]
Osman et al. (2004)	Peripheral blood	62	Nested RT-PCR	Monitoring recurrence	Positive predict value = 79%, negative predict value = 50%	[64]
Abrahamsson et al. (2017)	Peripheral blood	88	CellSearch System	Predict disease progression	Disease progression (*p* = 0.049)	[65]
Haga et al. (2020)	Peripheral blood	26	FISHMAN-R system	Predict disease progression	More CTC detected in progressive BC (*p* = 0.01)	[66]
Gradilone et al. (2010)	Peripheral blood	54	RT-PCR, CELLection Dynabeads	Monitoring recurrence	DFS (*p* < 0.001)	[67]
Nicolazzo et al. (2017)	Peripheral blood	54	RT-PCR, CELLection Dynabeads	Predict prognosis	DFS (*p* < 0.0001), CSS (*p* < 0.0001)	[68]
Busetto et al. (2017)	Peripheral blood	155	CellSearch System	Predict prognosis	TFR (*p* < 0.0001), TTP (*p* < 0.0001)	[69]
Nicolazzo et al. (2019)	Peripheral blood	102	CellSearch System	Predict prognosis	TFR (*p* < 0.001), TSR (*p* < 0.001), TTP (*p* < 0.001)	[70]
Fu et al. (2021)	Peripheral blood	48	Microfluidic-Assay System	Early risk stratification	More CTC detected in progressive BC (*p* = 0.024)	[71]
Winters et al. (2015)	Peripheral blood	31	CellSearch System	Predict treatment response	Evaluate chemotherapy response: CTC declined after chemotherapy	[72]
Nicolazzo et al. (2021)	Peripheral blood	20	CellSearch System, ScreenCell	Treatment monitoring	Serial evaluation of CTCs can guide treatment selection for suitable PD-L1 inhibitors after BCG failure	[73]
Anantharaman et al. (2016)	Peripheral blood	25	Algorithmic analysis	Predict treatment response	High PD-L1^+^ /CD45^−^ CTC burden and low burden of apoptotic CTCs had worse overall survival	[74]

*CTCs* Circulating tumor cells, *AUC* area under the receiver operating characteristics curve, *OS* Overall survival, *CSS* Cancer-specific survival, *RFS* Recurrence-free survival, *DFS* Disease free survival, *PFS* Progression free survival, *TFR* time to first recurrence, *TTP* time to progression, *TSR* time to second recurrence, *NAC* neoadjuvant chemotherapy, *BCG* Bacillus Calmette–Guérin, *PD-L1* Programmed death-ligand 1

### Circulating non-coding RNAs (ncRNAs) in BC

Blood ncRNAs can act as key functional components or regulatory molecules of gene expression in a variety of cancers, playing a role in tumorigenesis, cell differentiation, proliferation, inhibition of angiogenesis, metastasis, and apoptosis. Since miRNAs, lncRNAs, and circRNAs represent the most studied ncRNA types to date, we will briefly describe their roles in BC here. Table 2 summarizes the role of blood-based ncRNAs in BC. miRNAs are short ncRNAs, with an average length of 22 nucleotides. Normal and tumor cells secrete miRNAs into various body fluids, including plasma, urine, and vaginal secretions [75]. In blood, miRNAs can be bound to specific ribonucleoprotein complexes, platelets, or packaged in EVs, such as exosomes, to avoid degradation and to obtain higher stability [75]. There are two different strategies for cell-free miRNA studies in blood: (1) screening of differentially expressed miRNAs by microarray analysis or RNA sequencing, or (2) analysis of candidate miRNAs selected from published tumor tissue data. miRNAs are more rapidly biogenic and activated and highly stable, which may give miRNAs diagnostic and prognostic predictive aspects for BC potential.

**Table 2:** Studies regarding circulating ncRNAs in blood as potential biomarkers for bladder cancer

ncRNA	Authors (year)	Sample type	Significant miRNA expression	Study design	Detection method and reference method	Clinically relevant findings	Refs.
miRNA							
	Feng et al. (2014)	Plasma	miR-99a↓	• 50 patients • 50 controls	• RT-qPCR • RNU6	Diagnostic marker: no information concerning diagnostic accuracy	[76]
	Feng et al. (2014)	Plasma	• miR-19a↑	• 50 patients • 50 controls	• RT-qPCR • RNU6	Diagnostic marker: no information concerning diagnostic accuracy	[77]
	Adam et al. (2013)	Plasma	• miR-200b↑ • miR-33b↓ • miR-92b↓	• 20 patients • 18 controls	• RT-qPCR • RNU6	• 89% accuracy for detecting BC • 92% accuracy for distinguishing invasive BC from other cases • 100% accuracy for distinguishing MIBC from controls • 79% accuracy for three-way classification between MIBC, NIMBC, and controls	[78]
	Wang et al. (2019)	Serum	• miRNA‑373↑	• 55 patients • 45 controls	• RT-qPCR • RNU6	Diagnosis of BC (AUC = 0.847)	[79]
	Motawi et al. (2016)	Plasma	• miR-92a↓ • miR-100↓ • miR-143↓	• 70 patients • 62 controls	• RT-qPCR • RNU6	• Diagnosis of BC with miR-92a (AUC = 0.940, DS = 97.1%, DSp = 76.7%) • Diagnosis of BC with miR-100 (AUC = 0.820, DS = 90%, DSp = 66.7%) • Diagnosis of BC with miR-143 (AUC = 0.915, DS = 78.6%, DSp = 93.3%)	[80]
	Yang et al. (2015)	Serum	• miR-210↑	• 168 patients • 104 controls	• RT-qPCR • RNU6	• Diagnosis of BC (AUC = 0.898, DS = 97.6%, DSp = 69.2%) • Prognostic marker: monitor tumor dynamics	[81]
	Fang et al. (2016)	Plasma	• miR-205↑	• 89 patients • 56 controls	• RT-qPCR • RNU6	• Diagnosis of BC (AUC = 0.950, DS = 76.4%, DSp = 96.4%) • Differentiation of NIMBC from MIBC (AUC = 0.668, DS = 57.1%, DSp = 77.0%)	[82]
	Du et al. (2015)	Plasma	• miR-497↓ • miR-663b↑	• Discovery: pools of 10 patients and 10 controls • Validation: training set with 56 patients and 60 controls, and test set with 109 patients and 115 controls	• Discovery: TaqMan array • Validation: RT-qPCR	Diagnosis of cancer with the two combined miRNAs (AUC = 0.711, DS = 69.7%, DSp = 69.6%)	[83]
	Lian et al. (2018)	Serum	• miR-409-3p↓	• Discovery: 140 patients, 139 healthy controls • Validation: 140 patients, 139 controls	• Discovery: TaqMan array • Validation: RT-qPCR	Diagnostic marker: no information concerning diagnostic accuracy	[84]
	Jiang et al. (2015)	Serum	• miR-152↑ • miR-1486-3p↑ • miR-3187-3p↓ • miR-15b-5p↓ • miR-27a-3p↓ • miR-30a-5p ↓	• Discovery: pooled samples from 10 patients with NMIBC, 10 patients with MIBC, 10 healthy controls • Validation: training set 120 patients and 120 controls, and validation set 110 patients and 110 controls	• Discovery: sequencing • Validation: RT-qPCR	• Diagnosis of cancer with all miRNAs (AUC = 0.899, DS = 80%, DSp = 89%) • Differentiation of NIMBC from MIBC (AUC = 0.841, DS = 90%, DSp: 66.4%) • Prognostic marker set miR-152 and miR-3187-3p: recurrence-free survival for NIMBC, not for MIBC	[85]
	Jiang et al. (2016)	Serum	• miR-422a-3p (↑ in NMIBC, ↓in MIBC) • miR-486-3p↓ • miR-103a-3p (↑ in NMIBC, ↓in MIBC) • miR-27a-3p↓	• Discovery: pooled samples from 6 patients with NMIBC, 6 patients with MIBC, 6 healthy controls • Validation: training set 40 MIBC, 40 NMIBC, and 40 controls, and validation set 90 MIBC and 168 NMIBC	• Discovery: sequencing • Validation: RT-qPCR	• Diagnosis of cancer with the four combined miRNAs (training set: AUC = 0.894, validation set: AUC = 0.880) • Prognostic marker: miR-486-3p and miR-103a-3p were associated with OS of MIBC	[86]
	Usuba et al. (2019)	Serum	• miR-6087↓ • miR-6724-5p↑ • miR-3960↓ • miR-1343-5p↓ • miR-1185–1-3p↑ • miR-6831-5p↑ • miR-4695-5p↓	• Discovery: 196 patients with BC, 50 controls, 240 other types of cancers • Validation set: 196 patients with BC, 50 controls, 240 other types of cancers	• Discovery: TaqMan array • Validation: RT-qPCR	Diagnosis of cancer with the seven combined miRNAs (AUC = 0.97, DS = 95%, DSp = 87%)	[87]
LncRNA							
	Duan et al. (2016)	Serum	• lncRNA-MEG↓ • lncRNA- SNHG16↑ • lncRNA-MALAT1↑	• Discovery: pooled samples from 80 patients with BC and 80 healthy controls • Validation: training set 120 patients with BC, 68 patients with benign disease, 52 healthy controls, validation set 100 patients with BC, 52 patients with benign disease, 48 healthy controls	• RT-qPCR • GAPDH	• Diagnosis of cancer with three miRNAs (AUC = 0.899, DS = 80%, DSp = 89%) • Prognostic marker: low expression of MEG3 was associated with poor recurrence-free survival	[90]
	Luo et al. (2019)	Plasma	• lncRNA CASC11↑	• 89 patients • 62 controls	• RT-qPCR • RNU6	Diagnosis of BC (AUC = 0.899)	[91]
	Zhou et al. (2020)	Serum	• lncRNA LUCAT1↑	• 30 patients • 30 controls	• RT-qPCR • GAPDH	Diagnostic marker: no information concerning diagnostic accuracy	[92]
	Li et al. (2019)	Plasma	• lncRNA TUC338↑	• 56 patients • 56 controls	• RT-qPCR • 18S	Diagnosis of BC (AUC = 0.924)	[93]
	Zhang et al. (2020)	Serum	• lncRNA PCAT6↑	• 106 patients • 106 controls	• RT-qPCR • GAPDH	• Diagnosis of BC (AUC > 8.0) • Prognostic marker: high expression of lncRNA PCAT6 was associated with poor OS and PFS	[94]
circRNA							
	Pan et al. (2019)	Serum	• circ-FARSA↑ • circ-SHKBP1↑ • circ-BANP↑	• 70 patients • 70 controls	• RT-qPCR • β-actin	• Diagnosis of cancer with combined lncRNA UCA1 and circSHKBP1 (AUC = 0.804) • Combined circFARSA and circBANP distinguishing recurrent from non-recurrent patients (AUC = 0.737)	[95]
	Chi et al. (2019)	Serum	• hsa_circ_0000285↓	• 197 patients • 97 controls	• RT-qPCR • β-actin	• Diagnostic marker: no information concerning diagnostic accuracy • Treatment monitoring: low expression of hsa_circ_0000285 was associated with poor chemosensitivity • Prognostic marker: low expression of hsa_circ_0000285 was associated with poor survival rate	[96]
	Xu et al. (2018)	Blood	• circPTK2↑	• 40 patients • 40 controls	• RT-qPCR • GAPDH	Diagnostic marker: no information concerning diagnostic accuracy	[97]

*ncRNA* non-coding RNA, *miRNA* microRNA, *circRNA* circular RNA, *lncRNA* long non-coding RNA, *BC* bladder cancer, *MIBC* muscle-invasive bladder cancer, *NMIBC* non-muscle-invasive bladder cancer, *AUC* area under the receiver operating characteristics curve, *DS* diagnostic sensitivity, *DSp* diagnostic specificity, *RT-qPCR* reverse-transcription quantitative polymerase chain reaction. ↑ upregulated, ↓ downregulated

Many studies have explored the diagnostic, prognostic, and therapeutic potential of circulating miRNAs as potential biomarkers and therapeutic targets in BC. Feng et al. investigated plasma miR-19a and miR-99a in 100 patients with BC and healthy blood donors, respectively [76, 77]. As in tumor tissue, miR-19a expression was significantly increased in BC plasma, whereas miR-99a expression was significantly decreased, suggesting the potential of miR-19a and miR-99a as potential diagnostic markers for BC. Similarly, another study reported that plasma miR-200b expression was upregulated in patients with MIBC. In contrast, plasma miR-33b and miR-92b were downregulated in plasma from patients with BC and negatively correlated with pathological stage [78]. Recently, a study found that serum miRNA-373 levels were significantly higher in patients with BC compared with healthy controls and may serve as a potential diagnostic marker for BC (AUC was 0.847). In addition, miRNA-373 overexpression may promote BC cell proliferation, migration, and invasion [79].

Moreover, a study by Motawi et al. demonstrated that plasma miR-92a, miR-100, and miR-143 may be promising novel circulating biomarkers for clinical testing in BC [80]. The sensitivity and specificity of miR-92a calculated in this study were 97.1% and 76.7%, respectively, with an AUC value of 0.940. MiR-100 had a sensitivity and specificity of 90% and 66.7%, respectively, with an AUC value of 0.82. MiR-143 had a sensitivity and specificity of 78.6% and 93.3%, respectively, with an AUC value of 0.915. Similarly, another study observed that serum miR-210 expression was significantly elevated in patients with BC with a sensitivity of 97.6%, specificity of 69.2%, and AUC value of 0.898. Also, the authors found that serum miR-210 levels increased with increasing disease stage and grade. In addition, serum miR-210 expression was significantly lower in paired postoperative samples, whereas miR-210 expression was elevated in most patients with recurrent BC [81]. Similarly, a study found that plasma miR-205 levels were significantly higher in patients with BC than in normal controls, with a sensitivity of 76.4%, specificity of 96.4% and AUC: 0.950. Also, miR-205 could have the potential to be used as a biomarker to differentiate MIBC from NMIBC [82].

Using high-throughput TaqMan analysis, Du et al. identified miR-497 and miR-663b with significant differential expression by quantitative PCR for plasma miRNA analysis and two-stage validation of candidate miRNA expression. When integrating miR-497 and miR-663b, the AUC, sensitivity, and specificity were 0.711, 69.7%, and 69.6%, respectively [83]. Similarly, another study similarly used high-throughput TaqMan analysis to profile serum miRNAs to predict the risk of NMIBC. After validation in two independent cohorts (NMIBC: *n* = 280, control: *n* = 278), miR-409-3p was confirmed to be significantly associated with NMIBC [84].

Several groups developed a blood-based miRNA panel to improve the diagnostic efficacy of BC. Jiang et al. performed genome-wide serum miRNA analysis by MiSeq sequencing and established a panel of six miRNAs for the diagnosis of BC (miR-152, miR-3187-3p, miR-30a-5p, miR-27a-3p, miR-15b-5p, and miR-148b-3p). This miRNA model obtained high accuracy in this training set (sensitivity: 90.0%, specificity: 90.0%, AUC: 0.956) and was confirmed in the validation cohort (AUC: 0.899, sensitivity: 80.0%, specificity: 89.09%). Also, they found that serum miR-152 and miR-3187-3p expression was strongly associated with recurrence of NMIBC [85]. In the next step, they also defined a panel of four miRNAs (miR-422a-3p, miR-486-3p, miR-103a-3p, and miR-27A-3p) that predicted MIBC with high diagnostic accuracy (AUC: 0.89, sensitivity: 90.99%, specificity: 72.97%) and that was validated in an independent cohort (AUC: 0.88; sensitivity: 90.0%; specificity: 70.06%). Also, they found that miR-486-3p and miR-103a-3p were independently associated with OS [86]. A subsequent study found that a seven-miRNA panel (miR-6087, miR-6724-5p, miR-3960, miR-1343-5p, miR-1185–1-3p, miR-6831-5p, and miR-4695-5p) could be used as biomarkers for the specific and early detection of BC, with the highest accuracy for distinguishing BC from noncancerous tumors and other types of tumors (AUC: 0.97, sensitivity: 95%, specificity: 87%) [87].

In addition to miRNAs, several other non-coding RNAs, including lncRNAs and circRNAs, have been extensively studied and are associated with a variety of biological functions, including acting as microRNA sponges, RNA-binding proteins, regulating transcription, and encoding peptides [88, 89]. In view of BC, the utility of lncRNAs and circRNAs as prognostic, diagnostic, and therapeutic biomarkers was investigated [90–97]. Due to lack of space, their roles in BC were summarized in Table 2.

### Circulating tumor DNA (ctDNA) in BC

All tumor cells release DNA into the circulatory system, with cell-free DNA (cfDNA) constituting a major portion of released DNA circulating in the bloodstream [98]. Cancer cell-derived mutant cfDNA contains ctDNA, which consists of small nucleic acids released from necrotic or apoptotic tumor cells and circulating tumor cells, usually in the form of double-stranded fragments [99, 100]. The half-life of plasma ctDNA is short, estimated to be < 2 h, indicating that plasma ctDNA levels are in a state of dynamic and real-time change [101]. Although the half-life is relatively short, the rate of release of cancer DNA fragments is relatively constant owing to rapid cell turnover within tumors [102], providing real-time information on tumors in a longitudinal study, with multiple time points. ctDNA possesses several cancer-associated molecular features, such as single nucleotide mutations [103], altered methylation [104], and tumor-derived viral sequences [105], which may distinguish ctDNA from normal circulating cfDNA. There is a correlation between ctDNA levels and tumor size and staging, and analysis of ctDNA can help identify tumor-specific abnormalities that can be used in a highly specific detection strategy [106]. ctDNA levels also vary considerably with tumor staging, surgery, and chemotherapy [107]. Moreover, the collection of ctDNA from blood is noninvasive and can be useful for early cancer detection and identification of microscopic residual disease after treatment, in addition to cancer screening and prognosis prediction [108, 109]. ctDNA retains relatively intact genetic information when tumor cells enter the circulatory system after apoptosis or necrosis, clearly presenting all mutations throughout the patient’s tumor progression [108]. Therefore, the use of ctDNA could help address the issue of tumor heterogeneity, allowing the genotyping of multiple somatic aberrations across a wide genomic space in a single comprehensive assay [110]. Despite the pathological differences found between tissue and ctDNA-based mutation testing, ctDNA is frequently used to inform clinical decisions in the diagnosis of advanced disease, particularly in cases where biopsies are difficult to obtain or the primary location of the cancer is unknown [111]. Considering the heterogeneity of BC and the evolutionary pressure to intervene in treatment, ctDNA-based profiles are more reflective of advanced tumor burden than histology. However, ctDNA is challenging to detect, owing to its small percentage (sometimes < 0.01%), compared with non-tumorigenic cfDNA [112].

Currently, the main techniques for ctDNA detection in blood include quantitative polymerase chain reaction (qPCR) and digital polymerase chain reaction (dPCR), both of which allow the quantitative assessment of candidate genes and are rapid, cost-effective, and highly sensitive and specific. Additionally, both methods are suitable for the detection of certain simple mutations; however, the application of both methods is limited by the fact that they can only be used to monitor known genomic changes [113, 114]. To overcome this problem, next-generation sequencing (NGS)-based technologies are increasingly being applied to detect ctDNA, including a wide range of methods from small targeted panels to whole genome sequencing (WGS). None of these NGS techniques require any a priori molecular mutation knowledge, and they allow for the detection of known and unknown mutations, as well as hard-to-detect alterations, for example, gene fusions and copy number changes [102]. Additionally, NGS techniques can characterize personalized cancer genetic profiles, which could help in the development of personalized drugs. However, these techniques are expensive, time-consuming, and require more robust bioinformatics support [115]. In the era of precision oncology, the use of ctDNA analysis represents a paradigm shift in the use of genomic biomarkers, with considerable impact on clinical practice [116].

Compared with tissue-based tumor DNA analysis, plasma ctDNA is more convenient, easier and faster to obtain, and less invasive. Despite potential heterogeneity, tumor-specific genomic alterations detected in plasma ctDNA and tumor tissue have a fairly high concordance rate [117, 118]. These advantages facilitate the adoption of ctDNA as the companion diagnostic test to detect certain mutations required for the implementation of targeted therapies and for the serial surveillance of tumor progression [119]. An increase in ctDNA levels has been shown to occur prior to radiological progression [120, 121]. Additionally, the high somatic mutation rate in BC enhances the potential application of ctDNA, as fewer genes or targeted regions can provide the necessary information. Currently, ctDNA has a wide range of clinical applications in BC, including cancer screening, treatment response and drug resistance monitoring, and disease recurrence monitoring.

### ctDNA for BC screening and diagnosis

Several studies have shown that promoter methylation, leading to epigenetic inactivation of tumor suppressor genes, has diagnostic potential for BC. Analysis of preoperative plasma samples of patients with BC showed a significant association between ctDNA methylation status and aberrant methylation of tumor suppressor genes [122–126]. A study reported highly specific detection of methylated p16 DNA in the serum of patients with BC with sensitivity, specificity, and positive predictive values of 22.6, 95, and 98%, respectively [122]. Similarly, another study reported the presence of methylated p16 DNA sequences in the plasma of patients with BC and the absence of methylated ctDNA in healthy individuals [123]. These results suggest that methylated p16 can be used as a tumor marker for the diagnosis of BC. Additionally, a small cohort study involving 45 patients found that hypermethylation of serum ctDNA was detected in 59% of cases, and the hypermethylation of *APC*, *GSTP1*, or *TIG1* differentiated patients with BC from healthy individuals, with 80% sensitivity and 93% specificity [124]. Similarly, methylation of the promoter of tumor suppressor *CDH13* gene was observed in the serum of 30.7% of patients with BC, with a higher frequency in patients with advanced high-grade BC [125]. Moreover, a prospective multicenter study involving 227 patients with BC showed that serum ctDNA methylation is a useful noninvasive biomarker for differentiating patients with BC from healthy individuals (62% sensitivity, 89% specificity) [126]. Although the identification of aberrant gene promoter methylation could facilitate the detection of malignancies, its utility is still limited by the low concentrations of extracted ctDNA available for analysis. However, noninvasive testing for cancer-related alterations in DNA methylation is feasible, and this can be a source of information for liquid biopsies.

Other approaches involve identifying gene mutations rather than methylation changes in ctDNA. For examples, alterations in oncogenes are common in BC, with the most studied being *FGFR3*, *PIK3CA*, *ERBB2*, and *EGFR* [127]. Additionally, potential mutations were identified in the ctDNA of 51 patients with metastatic BC (including *RB1*, *CDKN2A*, and *ERBB2*), *MAPK*/*ERK* or *PI3K*/*AKT*/mTOR pathway-associated mutations, and chromatin remodeling-associated mutations [2]. Although most studies were limited by small sample sizes, all authors concluded that ctDNA analysis by liquid biopsy has potential applications as a prognostic biomarker in clinical settings.

### ctDNA as predictor of detecting recurrence and determining prognosis in BC

To date, the strongest evidence regarding the prognostic utility of liquid biopsy has been for ctDNA. CtDNA testing can be a valuable tool for early identification of disease recurrence after radical cystectomy (RC) or triple therapy for MIBC, which could facilitate early systemic treatment. In a previous study, ctDNA was collected at different timepoints before, during, and after treatment in 17 patients with MIBC who received neoadjuvant chemotherapy (NAC) and underwent surgery, and it was found that detection of ctDNA reflected persistence of disease, with a 83% sensitivity and 100% specificity for predicting recurrence [128]. Additionally, ctDNA levels were found to be significantly higher in patients who experienced metastatic recurrence or disease progression after cystectomy than in those who remained disease-free, with liquid biopsy results predating imaging evidence of recurrence by 101 days [129]. Moreover, additional ctDNA analysis after cystectomy has been shown to accurately predict metastatic recurrence, with 100% sensitivity and 98% specificity [130].

Others have used NGS to detect chromosomal breakpoints in 12 patients with NMIBC, and they subsequently designed droplet digital PCR (ddPCR) assays for these variants and found that somatic variants were detected in longitudinally collected plasma samples and that ctDNA levels decreased in patients after treatment, with high levels of ctDNA predicting disease progression. ctDNA can be detected early (before disease progression or recurrence, or before clinical manifestations). Moreover, 67% of patients with progressive disease have detectable levels of ctDNA in their plasma several months before evidence of clinical progression [131].

In addition, ctDNA can be widely detected in metastatic BC and has outstanding clinical application value [2, 131, 132]. In a recent study in a large mUC cohort comparing blood-based “liquid” biopsies with patient-matched tumor tissue, ctDNA provided a more representative genomic snapshot of disease. ctDNA analysis can identify genetic alterations such as *FGFR3*, *ERCC2*, *ERBB2*, and *TMB*, which were previously proposed as biomarkers of BC treatment response. Cost-effective and minimally invasive approaches to identify them will enable biomarker-driven stratification of patients in clinical trials, as well as real-world application of precision oncology [118].

The analysis of tumor-associated genomic alterations in ctDNA assays reflects an important approach for biomarker discovery and prognosis in patients with advanced malignancies [133–135]. A previous study reported disease progression using ddPCR to determine ctDNA for *FGFR3* and *PI3KCA* hotspot mutations. It was found that tumor *FGFR3* and *PI3KCA* mutations were significantly associated with subsequent disease recurrence, and that the expression of genes with multiple hotspot mutations could improve the prognostic monitoring performance of ctDNA [133]. Sequential ctDNA analysis can monitor dynamic changes in the frequency of genomically altered variant alleles and can predict disease progression and guide precise medication administration in patients with advanced BC [134]. In a recent retrospective study, ctDNA was found to identify clinically relevant molecular abnormalities, particularly, alterations in the BRCA-1 DNA repair-associated (*BRCA1*) and Raf-1 proto-oncogene (*RAF1*) genes appeared to be negatively associated with clinical outcomes [134].

The association between specific ctDNA genomic mutations and BC prognosis provides a new reference and basis for personalized BC treatment [136]. An association between the number of genomic alterations detected in ctDNA and the rate of disease control after receiving immunotherapy has also been suggested. In a recent randomized phase III trial involving 581 patients with uroepithelial carcinoma who had undergone surgery, ctDNA-positive patients in the atezolizumab treatment group were found to have improved disease-free survival and overall survival compared with the observation group [137]. Another study examined mutations in plasma ctDNA in 82 patients with NMIBC who underwent transurethral resection of the urinary bladder (TUR) and received immunotherapy, and found that higher somatic variants were independent predictors of recurrence after immunotherapy after bladder TUR. These findings suggest that ctDNA analysis based on targeted sequencing is a promising method for predicting disease recurrence in patients with NMIBC undergoing bladder TUR and immunotherapy [138].

### ctDNA as predictor of monitoring response to treatment

Quantitative changes in ctDNA levels have shown potential as an early indicator of treatment efficacy. Monitoring ctDNA scores during treatment has been reported to be useful in predicting treatment response in metastatic uroepithelial carcinoma. Treatment-responsive patients exhibited rapidly decreasing ctDNA scores, whereas non-response patients exhibited stable or increasing scores, indicating that ctDNA could be used to identify responsive patients prior to imaging [139]. Moreover, dynamic changes in ctDNA during chemotherapy in patients with BC showed a superior association with patient prognosis [140]. In contrast, patients with ctDNA clearance indicated a response to chemotherapy, and could provide additional cycles of chemotherapy before cystectomy [130]. A recent study showed that ctDNA can be used as a marker for MRD to predict response to adjuvant immunotherapy in patients with BC [137]. Overall, ctDNA analysis could be used to detect MRD and monitor treatment effects at early timepoints when patients can benefit from change in treatment regimen.

Additionally, an association between the number of genomic alterations detected in ctDNA and response to immunotherapy has been observed [134, 141–143]. Changes in the frequency of ctDNA variant alleles early in treatment were found to identify checkpoint inhibitor monotherapy non-responders, thus helping to adjust treatment decisions. ctDNA levels are a predictor of long-term benefits of BC immunotherapy [142]. A previous study showed that sequential ctDNA analysis can be used to monitor dynamic changes in the frequency of genomically altered variant alleles and to predict treatment response. Serial ctDNA testing is a promising option for dynamically monitoring ICI treatment effects, predicting outcomes, and guiding adaptive treatment modalities [134]. One or more genomic alterations in ctDNA, mainly *TP53*, *TERT*, and *BRCA1*/*BRCA2*, were detected in 95% and 100% of patients before and after treatment with ICI, respectively, and genomic alterations in these oncogenic drivers were strongly associated with tumor resistance in advanced uroepithelial carcinoma [143]. Studies regarding blood-based ctDNAs as potential biomarkers for BC were summarized in Table 3.

**Table 3:** Studies regarding circulating DNAs in blood as potential biomarkers for bladder cancer

Authors (year)	Sample type	No. of patients	Laboratory technique	Clinical application	Detection rate	Refs.
Valenzuela et al. (2002)	Serum	135	Methylation-specific PCR	Diagnostic biomarker	AUC = 95%, sensitivity = 22.6%, specificity = 98%	[122]
Domínguez et al. (2002)	Plasma	27	5–4520 kit, QIAamp Blood kit, PCR	Diagnostic biomarker	Detected in 40% patients	[123]
Ellinger et al. (2008)	Serum	45	Restriction endonuclease-based assay, qRT-PCR	Increase the accuracy of the diagnosis of BC	Sensitivity = 80%, specificity = 93%	[124]
Lin et al. (2011)	Serum	168	Methylation-specific PCR	Diagnostic biomarker	Detected in 30.7% patients, higher in advanced BC	[125]
Hauser et al. (2013)	Serum	227	Methylation-specific PCR	Discrimination of patients with BC from healthy individuals	Sensitivity = 62%, specificity = 89%	[126]
Vandekerkhove et al. (2017)	Plasma	51	Targeted and exome sequencing	Revealing aggressive mutations in metastatic BC	95% of patients harboring deleterious alterations	[2]
Patel et al. (2017)	Plasma	17	TAm-Seq, WGS	Monitoring recurrence	Positive predict value = 100%, negative predict value = 85.7%	[128]
Birkenkamp-Demtröder et al. (2018)	Plasma	60	WES, ddPCR	Monitoring recurrence	Earlier recurrence detection compared with imaging	[129]
Christensen et al. (2019)	Plasma	68	WES, ultra-deep sequencing	• Predict metastatic recurrence • Monitoring of therapeutic efficacy	• Sensitivity = 100%, specificity = 98%; • Changes in ctDNA during chemotherapy in high-risk patients correlated with disease recurrence (*p* = 0.023)	[130]
Birkenkamp-Demtröder et al. (2016)	Plasma	12	NGS, ddPCR	Predicts disease progression and residual disease	Disease progression (*p* = 0.032)	[131]
Vandekerkhove et al. (2021)	Plasma	104	WES, QIAGEN DNeasy Blood and Tissue Kit	Predict prognosis	OS (*p* = 0.01), PFS (*p* = 0.02)	[118]
Shohdy et al. (2022)	Plasma	182	NGS, WES	Predict disease progression	OS (*p* = 0.03)	[134]
Grivas et al. (2019)	Blood	124	Exon sequencing	Predict prognosis	OS (*p* = 0.07), FFS (*p* = 0.016)	[135]
Powles et al. (2021)	Plasma	581	WES, multiplex PCR	• Predict recurrence • Predict treatment response	• DFS (*p* < 0.0001) • ctDNA can be used as a marker for MRD to predict response to adjuvant immunotherapy	[137]
Zhang et al. (2021)	Plasma	82	Targeted sequencing	Predict prognosis	DFS (*p* = 0.0146)	[138]
Sundahl et al. (2019)	Blood	9	RT-PCR	Response monitoring	Predicting treatment response in metastatic uroepithelial carcinoma prior to imaging	[139]
Khagi et al. (2017)	Blood	69	NGS	Predict treatment response	ctDNA-determined hypermutated states predict improved response, PFS, and OS after checkpoint inhibitor therapy	[141]
Raja et al. (2018)	Plasma	29	Targeted sequencing	Predict treatment response	Changes in the frequency of ctDNA variant alleles early in treatment were found to identify checkpoint inhibitor monotherapy non-responders	[142]
Ravi et al. (2022)	Blood	45	NGS	Treatment monitoring	Detection of one or more genomic alterations in ctDNA before and after ICI treatment is associated with tumor resistance in advanced uroepithelial carcinoma	[143]

*ctDNA* circulating tumor deoxyribonucleic acid, *AUC* area under the receiver operating characteristics curve, *WGS* whole genome sequencing, *WES* whole-exome sequencing, *NGS* next-generation sequencing, *PCR* polymerase chain reaction, *ddPCR* droplet digital PCR, *OS* overall survival, *PFS* progression free survival, *DFS* disease free survival, *FFS* failure-free survival, *MRD* minimal residual disease, *ICIs* immune checkpoint inhibitors

### Exosomes

Exosomes are a subtype of a broad class of EVs, ranging from 40 to 100 nm in diameter, which are thought to be lipid bilayer membrane vesicles actively released by most cells and capable of stable circulation in body fluids [144, 145]. Exosomes are released by both normal and tumor cells, and are present in various body fluids, such as urine, plasma, saliva, and tears [146]. The biogenesis and release of exosomes is a complex multistep process involving plasma membrane invagination, formation of multivesicular bodies, and exosome secretion. Several studies have shown that donor cell-derived bioactive molecules are enriched in exosomes, indicating the key role of exosomes in the exchange of genetic information [147]. Exosomes carry specific proteins (CD9, CD81, CD63, ALIX, TSG101, and HSP70), nucleic acids (DNA, miRNA, lncRNA, and circRNA), lipids, and metabolites with cancer information, making them potential cancer diagnostic biomarkers. Moreover, their specific cup-like features can be identified by electron microscopy [147–149]. Moreover, their specific cup-like features can also be identified by electron microscopy [150].

Exosomes play key roles in many pathophysiological processes, including immune responses, cardiovascular diseases, pregnancy disorders, and cancer [151, 152]. Exosomes are involved in EMT, angiogenesis, invasion and metastasis, evasion of immune surveillance, and drug resistance during tumorigenesis and progression, with promising clinical applications. Additionally, exosomes can be designed to deliver different therapeutic loads, including short interfering RNAs, antisense oligonucleotides, chemotherapeutic agents, and immunomodulators, indicating their potential application in tumor therapy [147]. In addition to their therapeutic potential, exosome-based liquid biopsies highlight their potential application in early cancer diagnosis, progression, prognosis, and response to therapy [153, 154]. Current reviews on BC exosomes focus on miRNAs, lncRNAs, and circRNAs.

Exosomes possess potential applications in precision tumor therapy, owing to their unique biological properties. However, the isolation and enrichment of exosomes from complex biological components is crucial for basic research and clinical translation. To date, some methods have been developed for exosome isolation and enrichment, with varying output and purity [155, 156]. Traditional separation methods include ultracentrifugation, size-exclusion chromatography, sedimentation techniques, and density gradient/buffered centrifugation. Recently, novel techniques, such as immunoaffinity assays, lipid-based separation techniques, microbeads/microfluidic chips, and thermophoresis, have made the enrichment of exosomes fast and easy. However, there is need to standardize the classification and extraction methods of exosomes from different body fluids to facilitate clinical use of exosomes.

### Exosomes in BC diagnosis

Serum exosomes have been examined as noninvasive stable and sensitive biomarkers for the early diagnosis of BC. Exosomes reveal information about living tumor cells, and possess promising opportunities for the early detection of lesions [157–161]. Elsharkawi et al. observed an increase in the serum exosome levels of patients with BC with increasing stages of the disease. Moreover, patients with BC were successfully differentiated from healthy individuals on the basis of serum BC exosome levels, with 100% specificity and 82.4% sensitivity [160]. Similarly, Wang et al. observed an upregulation in exosomal lncRNA H19 levels in patients with BC compared with healthy subjects, indicating that exosomal lncRNA H19 could be a novel minimally invasive diagnostic biomarker [157]. Moreover, a previous study showed that hypoxic BC cells secreted lncRNA-rich uroepithelial carcinoma-associated 1 (UCA1) exosomes to remodel the tumor microenvironment and promote tumor growth and progression, indicating that serum exosomal lncRNA UCA1 could be a potential diagnostic biomarker of BC [159]. Furthermore, Zheng et al. found that exosomal lncRNA PTENP1 levels could be used to successfully differentiate patients with BC from healthy individuals, with an AUC value of 0.743 [161].

Some studies have also explored the combined application of exosomal non-coding RNAs in the diagnosis of BC. For example, a diagnostic model based on the combination of three serum exosomal lncRNAs (PCAT-1, UBC1, and SNHG16) was highly accurate for the early diagnosis of BC, with an AUC of 0.826, sensitivity of 80%, and specificity of 75%, which were significantly higher than the results of urine cytology [158].

### Exosomes in BC prognosis

Recently, exosomal miRNAs have been found to play important roles in tumorigenesis and progression [162–164]. Exosomal miRNAs regulate communication and multiple signaling pathways between BC cells and other cells, and cancer cell-derived exosomes have been associated with BC progression and metastasis through enriched specific miRNAs [163]. Exosomal miR-21 from BC cells has been reported to promote BC cell invasion and migration by polarizing the *PI3K*/*AKT *signaling pathway in tumor-associated macrophages [165]. Additionally, the exosome-based miR-139-5p delivery system was found to help inhibit the proliferation, migration, and invasive potential of BC in vitro and in vivo, presenting potential innovative options to curb carcinogenesis [166].

Several studies have shown that blood-based exosomes can provide novel noninvasive prognostic biomarkers for BC. Yin et al. found that miR-663b levels were elevated in plasma exosomes of patients with BC compared with healthy controls. Exosomal miR-663b from BC cells promotes BC cell proliferation and tumor progression by mediating the function of *ERF* [167]. Yan et al. observed higher levels of miR-4644 in plasma exosomes from patients with BC compared with patients without BC; additionally, exosomal miR-4644 directly targets and downregulates the expression of *UBIAD1*-containing protein 1 (UBIA prenyltransferase domain-containing protein 1) and promotes BC cell proliferation, migration and invasion [168]. Conversely, two recent studies demonstrated that both miR-375-3p and miR-133b were significantly lower in BC serum, and inhibited cell proliferation and metastasis, but promoted apoptosis in both in vivo and in vitro models, serving as suppressors and potential therapeutic targets of BC [169, 170]. Furthermore, Sabo et al. confirmed that low expression of miR-185-5p and miR-106a-5p, or high expression of miR-10b-5p, in exosomes from BC serum was significantly associated with shorter survival [171].

Additionally, studies have shown that exosomal lncRNAs play a crucial role in cell proliferation and invasion, lymphangiogenesis, drug resistance, and prognostic assessment in BC [158, 161, 172–177]. A research group found that cancer-associated fibroblast-derived exosome-mediated LINC00355 not only promotes cisplatin resistance in BC cells, but is also involved in regulating BC cell proliferation and invasion [172, 173]. Exosome-mediated lncRNA LINC01133 can inhibit BC suppression of cell viability, proliferation, migration, and invasion ability by regulating the Wnt signaling pathway, which may provide a novel target for BC therapy [174]. High expression of lncRNA UBC1 in serum exosomes is associated with a high recurrence rate of NMIBC, and is an independent risk factor for recurrence-free survival in NMIBC [158]. Zheng et al. found that exosomes from normal cells transferred *PTENP1* into BC cells and reduced the invasive and migratory capacity of the cells in vitro and reduced BC progression both in vivo and in vitro [161].

The prognosis of patients with BC with lymph node metastases is extremely poor in clinical practice. It has been reported that exosomes can act as a vehicle of communication between the lymphatic system and BC, potentially remodeling the lymphatic system laterally by transferring epigenetic and genetic information [178]. Chen et al. identified an exosomal lncRNA, called lymph node metastasis-associated transcript 2 (LNMAT2), which was significantly elevated in BC serum samples [177]. Additionally, higher LNMAT2 expression levels were detected in serum exosomes of patients with BC with lymph node metastasis compared with patients without lymph node metastasis. Moreover, exosomes secreted by BC cells are internalized by human lymphatic endothelial cells, which triggers the upregulation of *Prox1*-expressing exosomes through *hnRNPA2B1* recruitment and increases *H3K4* trimethylation of the *Prox1* promoter, leading to lymphangiogenesis and lymph node metastasis in BC cells [176]. Furthermore, serum exosome *LNMAT2* overexpression is also associated with shorter OS in patients with BC [177].

Exosomal circRNAs are abundant and stably present in tumors, and are important for intercellular communication [179]. Recent studies using high-throughput sequencing have revealed that several exosomal circRNAs can serve as biomarkers for cancer diagnosis, invasion, and drug resistance [180, 181]. It was found that blood exosome-derived circTRPS1 from patients with BC can regulate intracellular activity homeostasis and CD8^+^ T cell depletion through the circTRPS1/miR141-3p/GLS1 axis, enhance BC cell proliferation and invasiveness, and impair antitumor immune function in the BC microenvironment [182]. Recent studies found that circPRMT5 is upregulated in serum exosomes of patients with BC, and plays a key role in the EMT and/or invasiveness of BC cells; moreover, high expression of circPRMT5 is positively correlated with metastasis and/or poorer survival in patients with BC, making it a potential therapeutic target for patients with BC [183].

Furthermore, it was found that the CT10 regulator kinase (CRK) promoted the expression of *ErbB2* in BC cells, and these tumor proteins were transferred via exosomes to receptor cells in target organs, contributing to the distant metastasis of BC. These findings indicate that CRK intervention with exosomal *ErbB2* blockade may be an effective therapeutic strategy for patients with advanced and metastatic BC with *ErbB2* overexpression [184]. Studies regarding blood-based exosomes as potential biomarkers for BC were summarized in Table 4.

**Table 4:** Studies regarding blood-based exosomes as potential biomarkers for bladder cancer

Authors (year)	Sample type	Functional component	No. of patients	Laboratory technique	Clinical application	Detection rate	Refs.
Wang et al. (2018)	Serum	H19	104	ExoQuick exosome purification kit	Diagnostic biomarker	AUC = 85.1%, sensitivity = 74.07%, specificity = 78.8%	[157]
Zhang et al. (2019)	Serum	Three-lncRNA panel (PCAT-1, UBC1, and SNHG16)	520	ExoQuick exosome purification kit	Diagnostic biomarker	AUC = 0.857 in training set, and 0.826 in validation set	[158]
Xue et al. (2017)	Serum	lncRNA-UCA1	60	ExoQuick exosome purification kit	Diagnostic biomarker	AUC = 87.83%, sensitivity = 80%, specificity = 83.33%	[159]
Elsharkawi et al. (2019)	Serum	Tumor-derived exosomes	82	ExoQuantTM overall exosome capture and quantification assay kit, ELISA	Diagnostic biomarker	Sensitivity = 82.4%, specificity = 100%	[160]
Zheng et al. (2018)	Plasma	lncRNA-PTENP1	110	ExoQuick exosome purification kit	Diagnostic biomarker, predict disease progression	AUC = 74.3%, sensitivity = 65.4%, specificity = 84.2%, predict disease progression (*p* < 0.05)	[161]
Yin et al. (2020)	Plasma	miR-663b	122	ExoQuick‐TC Exosome Precipitation Solution	Predict disease progression	Disease progression (*p* < 0.05)	[167]
Yan et al. (2020)	Plasma	LINC00355	57	miRNA microarray	Predict disease progression	Disease progression (*p* < 0.05)	[168]
Cai et al. (2020)	Serum	miR-133b	11	Total exosome isolation reagent	Predict disease progression	Disease progression (*p* < 0.05)	[170]
Sabo et al. (2020)	Plasma	miR-126-3p	93	Deep sequencing	Predict prognosis	OS (*p* < 0.05)	[171]
Zhang et al. (2019)	Serum	LINC00355	520	ExoQuick exosome purification kit	Predict prognosis	RFS (*p* = 0.01)	
Chen et al. (2020)	Serum	circRNA hsa_circ_0051443	592	FRET, CD spectroscopy	Predict prognosis	OS (*p* < 0.05)	[181]
Yang et al. (2022)	Serum	circTRPS1	90	ExoQuick Exosome Precipitation Solution	Predict prognosis	OS (*p* = 0.01)	[182]
Chen et al. (2018)	Serum	circRNA-PRMT5	119	Electron microscopy	Predict prognosis	OS (*p* = 0.028)	[183]

*miRNA* microRNA, *circRNA* circular RNA, *lncRNA* long non-coding RNA, *AUC* area under the receiver operating characteristics curve, *ELISA* enzyme linked immunosorbent assay, *OS* overall survival, *RFS* recurrence-free survival

### Blood-based metabolomics and proteomics

Among the molecular and analytical techniques used to identify biomarkers, modern metabolomics technologies have made significant advances in characterizing and differentiating patients with BC from control subjects, identifying signature metabolites, and revealing disease biology and potential therapeutic targets. Metabolomics attempts to use the metabolic profile of cancer to assess disease risk, enabling real-time documentation and monitoring of disease stages, prediction of prognosis, recurrence or progression, and assessment of response or resistance to therapy [185]. Metabolomics is a comprehensive analysis of small molecule metabolites and can provide critical information on cancer stage [186, 187].

Metabolomics is a functional readout of tissue biochemistry that better reflects phenotypic status than other methods, such as transcriptomics and proteomics. There is increasing evidence that alterations in tumor metabolism may lead to local immunosuppression in the tumor microenvironment. Recently, metabolomic techniques are increasingly being utilized to identify potential biomarkers for cancer detection and monitoring. Serum/plasma is usually unaffected by exogenous factors and therefore varies little between individuals [188]. The analysis of sera from patients with BC allows the quantification of surface adhesion molecules, proteins, lipids, and amino acids. The chromatographic properties and mass spectra obtained provide features that can be compared with known metabolomics libraries, resulting in a “fingerprint” that becomes a biomarker. Metabolomics can provide a better understanding of the biological mechanisms of each tumor phenotype, thus promoting precision medicine and personalizing treatment for patients with different cancer types.

The main techniques used in metabolomics include gas chromatography–mass spectrometry (GC–MS), liquid chromatography–mass spectrometry (LC–MS), and nuclear magnetic resonance (NMR) methods for the detection of compounds in any biological fluid or cellular/tissue liquid-phase extracts [189, 190]. GC–MS and LC–MS are the two most widely used analytical techniques in metabolomics and can be used to identify compounds with a unique quality of several thousand features [191]. Among them, LC–MS-based metabolomics has become increasingly popular, owing to the high coverage of metabolites, further improving detection sensitivity and data reliability in cancer metabolomic studies [192]. NMR is a versatile method that can be used for biological samples in liquid, solid, or gaseous form without prior processing [193]. Other techniques for metabolite analysis are more limited, such as matrix-assisted laser desorption/ionization mass spectrometry imaging and magnetic resonance spectroscopy imaging. The increased sensitivity, specificity, and development of metabolomics-related technologies may help to identify good metabolic biomarkers, which can be applied in clinical settings.

Despite considerable improvements in genomics- and metabolomics-based strategies, the importance of proteomic-based analysis in liquid biopsies for cancer cannot be overemphasized. Recent advances in clinical proteomic techniques offer promising opportunities for the study of proteins in plasma/serum [194]. Since all tissues of the body are perfused by blood, each cell leaves a “trace” of its constituent proteins and other compounds in the circulatory system. Blood-based proteomics studies and links protein expression profiles to specific disease phenotypes, and identifies potential biomarkers reflecting the physiological or pathological state of the body, which may aid early disease detection.

Recently, separation techniques, such as electrophoresis, reversed-phase liquid chromatography, ion exchange and size-exclusion chromatography, and immune-enrichment or depletion methods, have been used to isolate or concentrate proteins or peptides, followed by mass spectrometry in combination with advanced computer software, making it possible to accurately identify, characterize, and quantify proteomic data [195]. Mass spectrometry-based proteomics can be divided into data-intensive discovery-based proteomics and targeted proteomics. The former allows unbiased analysis of the proteome on a global scale, whereas the latter provides highly specific quantitative detection of selected candidates in biological samples. To date, blood-based proteomics studies have provided considerable information on BC-associated proteomic changes, providing a better understanding of the intrinsic changes in the proteome of the blood during BC.

### Role of blood-based metabolomics and proteomics in BC diagnosis

Metabolomics and proteomics may help identify biomarkers for the diagnosis of BC. Serum is recognized for biomarker studies because it is constantly perfused in tissues and may pick up disturbed metabolites and proteins secreted and shed from cells and tissues. A previous study showed that patients with BC had lower serum levels of citric acid, glycine, isoleucine/leucine, lactate, and tyrosine, and higher levels of lipids and glucose compared with healthy individuals [196]. Additionally, NMR spectroscopy-based serum metabolomics showed that a combination of dimethylamine (DMA), glutamine, and malonic acid accurately isolated high-grade BC from low-grade BC, with good external validation (96% sensitivity and 94% specificity) [197]. Moreover, metabolites involved in malignant proliferation, immune escape, differentiation, apoptosis, and invasion of cancer cells were identified by ultra-high performance liquid chromatography (UHPLC) combined with Q-TOF mass spectrometry, and three combinations of serum metabolites, namely inosine, N1acetyl-N2-formyl-5-methoxykynurenine, and PS (O-18:0/0:0), showed good properties in predicting high-level BC [198].

Compared to urine analysis, proteomic studies using blood are rare. However, it was found that the proteomic pattern of serum samples from patients with BC versus controls was an effective screening tool for identifying diagnostic BC, with 96.4% sensitivity and 86.5% specificity, even in low stage low grade tumors [199]. Bansal et al. analyzed 80 serum samples from healthy individuals and patients with low-grade and high-grade BC using bidirectional gel electrophoresis, and identified five differentially expressed proteins, including S100A8 and S100A9, which accurately distinguished between BC (low-grade and high-grade) and healthy controls, with an AUC value of 0.946 [200]. Similarly, abnormally expressed serum levels of S100A4, S100A8, S100A9, CA-I, and annexin V proteins were found to serve to be effective protein biomarkers for BC diagnosis based on the analysis of 160 serum samples from 52 healthy individuals, 55 presurgical, and 53 postsurgical patients with BC [201].

### Role of blood-based metabolomics and proteomics in BC disease progression

Metabolomics and proteomics can help identify potential biomarkers of high aggressiveness in patients with BC [202–204]. Certain metabolic pathways may be associated with the regulation of chemosensitivity and immunotherapy in BC and may serve as potential biomarkers for identifying high-risk patients [205, 206]. A previous metabolomic showed that a set of genetic features, specifically *COMT*, *IYD*, and *TTL*, play potentially important roles in the progression of BC in smokers [207].

Minami et al. demonstrated that tumor-associated proteins S100A8 and S100A9 are associated with BC staging, grading and CSS, and S100A8 expression is an important predictor of BC recurrence [208]. Similarly, a total of nine differentially expressed glycoproteins in another study, including haptoglobin, transferrin, and IgM glyco-variants, or various cleavage products of fibrinogen and complement C3b, may be promising in the monitoring of BC disease progression [209]. Studies regarding blood-based metabolomics and proteomics as potential biomarkers for BC were summarized in Table 5.

**Table 5:** Studies regarding circulating metabolomics and proteomics in blood as potential biomarkers for bladder cancer

Authors (year)	Sample type	No. of patients	Laboratory technique	Clinical application	Detection rate	Refs.
Cao et al. (2012)	Serum	112	^1^H NMR measurements	Discrimination of patients with BC from healthy individuals	Detected abnormal serum metabolic profiles in 100% of patients	[196]
Bansal et al. (2013)	Serum	99	^1^H NMR measurements	Diagnostic biomarker	Sensitivity = 94%, specificity = 97%	[197]
Tan et al. (2017)	Serum	172	UHPLC coupled with Q-TOF MS	Diagnostic biomarker	AUC = 0.961	[198]
Schwamborn et al. (2009)	Serum	248	MALDI-TOF–MS	Diagnostic biomarker	Sensitivity = 96.4%, specificity = 86.5%	[199]
Bansal et al. (2014)	Serum	90	MALDI-TOF–MS	Diagnostic biomarker	AUC = 0.946 (S100A8 and S100A9)	[200]
Bansal et al. (2016)	Serum	160	ELISA, MARS	Diagnostic biomarker	AUC = 0.957 (S100A9)	[201]
Amara et al. (2019)	Serum	87	LC–MS	Predict disease progression	OS (*p* = 0.0065)	[207]
Minami et al. (2010)	Serum	2	Reversed-phase high-performance liquid chromatography	Predict prognosis	RFS (*p* = 0.026), CSS (*p* = 0.041)	[208]
Lemańska-Perek et al. (2019)	Plasma	6	MALDI-TOF–MS	Predict disease progression	Increasing abundance in progressing BC	[209]

*AUC* area under the receiver operating characteristics curve, *ELISA* enzyme linked immunosorbent assay, *OS* overall survival, *RFS* recurrence-free survival, *CSS* cancer-specific survival, *NMR* nuclear magnetic resonance, *LC–MS* liquid chromatography–mass spectrometry, *Q-TOF MS* quadrupole time of flight mass spectrometry, *UHPLC* ultra-high performance liquid chromatography, *MALDI-TOF–MS* matrix-assisted laser desorption/ionization time of flight mass spectrometry

### Challenges facing liquid biopsy in BC

Liquid biopsies represent a promising strategy in biomarker research. Despite the considerable potential implications, a range of technical, biological, and clinical challenges need to be addressed before liquid biomarkers can be adopted in clinical practice. Limitations such as the lack of standardized assays and the high cost of liquid biopsies hinder the potential translation of liquid biomarkers into clinical practice. Additionally, most studies evaluating liquid biopsy techniques have been limited by small sample sizes, indicating the need for large-scale higher-quality studies to validate previous findings before routine clinical application.

CTC analysis can provide unique insights into tumor heterogeneity; however, CTC count during early-stage disease is low and may be undetected. Moreover, different tumor regions in a single patient may not show the same CTC propensity. Therefore, it is unclear whether CTC analysis could cause potential biological bias in intratumor heterogeneity. Although several techniques for CTC detection have emerged, their sensitivity and specificity still require further improvement. High-throughput sequencing can facilitate CTC-based multiomics studies, which can further benefit clinical applications, but can also contribute to the difficulty and complexity of data analysis [210].

The advantage of ctDNA analysis over CTC detection is that plasma/serum samples can be stored for a longer period of time and are easily collected before final analysis, making ctDNA an attractive option for multicenter studies. Currently, ctDNA analysis is a major method for assessing BC heterogeneity, without sampling bias of tissue biopsy. However, most studies on BC liquid biopsies have focused on patients with advanced disease. The use of ctDNA for early detection may be highly limited due to the low detection rate of early cancers, and therefore there is a need to develop techniques with higher sensitivity and to obtain sufficient sample volumes to detect trace amounts of ctDNA. Additionally, the sensitivity, specificity, and reproducibility of ctDNA assays, and the optimization and standardization of preanalytical conditions, require further studies [211].

Exosomes are a novel means of intercellular information exchange and play an important role in the tumor microenvironment. However, the heterogeneity and number of exosomes in body fluids may be a drawback to their utility as biomarkers, as these may yield false negatives or positives in cancer diagnosis. Moreover, further studies are necessary to develop and optimize methods for loading bioactive substances into exosomes. Additionally, extensive studies on the safety, targeting ability, and efficacy of exosomes are necessary to facilitate the successful application of exosomes as targets or drug delivery tools in BC.

Before fully evaluating cfRNA for clinical applications, the sensitivity of cfRNA for cancer detection in early-stage cancer needs to be determined. This is because cfRNA is unstable and highly fragmented. Targeted assays such as hybridization capture or amplicon sequencing should be used, which will allow for more sensitive quantification of cfRNA.

**Fig. 3:**
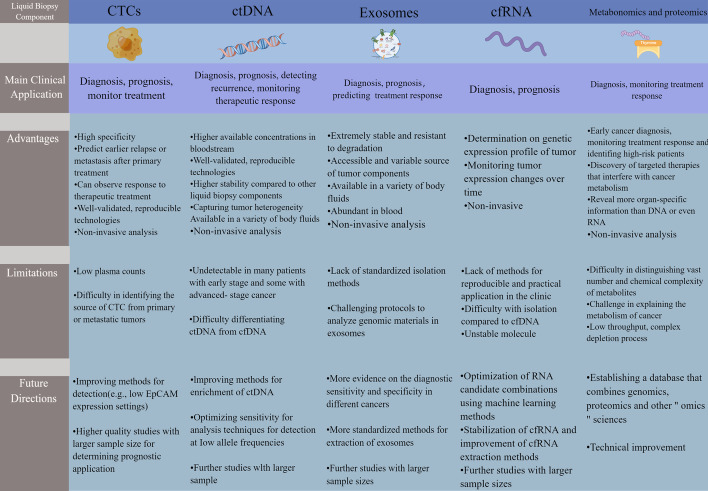
Comparison of the major liquid biopsy methods in bladder cancer and their main advantages, limitations, clinical applications, and future directions

Furthermore, BC metabolic profiles are mainly characterized by alterations in metabolites associated with energy metabolic pathways, amino acid metabolism, and fatty acid metabolism, which are important for cell proliferation and maintenance of cellular redox homeostasis. However, the lack of standardized sample collection methods, variations in metabolite analysis methods, environmental stress, and food intake of patients severely influence metabolome composition, all of which contribute to differences in metabolomic profiles obtained from different laboratories. The analysis of changes in serum protein profiles in patients with BC is an emerging area of liquid biopsy that can reveal complex interactions between tumor tissue and the surrounding vascular microenvironment. However, additional clinical trials are still needed to validate previous proteomic data. Despite these challenges, metabolomics and proteomics show great clinical promise. Figure 3 provides a summary of the main advantages, disadvantages, and clinical applications of liquid biopsy components represented by CTCs, ctDNA, cfRNA, exosomes, and metabolomics and proteomics.

## Conclusion and future perspectives

### Management strategy for patients with BC

Despite the high accuracy of cystoscopy and pathology biopsy in detecting BC, there are multiple limitations to their application for screening BC, including complications associated with invasive biopsy, patient anxiety, and financial burden. Although not currently considered a standard tool for the confirmation and diagnosis of BC, liquid biopsy could be a promising and effective alternative to traditional invasive sampling methods, especially in cases where tissue samples are not available. Additionally, it may be reasonable to defer invasive cystoscopy in patients with low risk of recurrence on the basis of preliminary liquid biopsy, which could reduce patients’ pain. The use of circulating biomarkers and liquid biopsies in real-time assessment of BC during treatment could provide useful information for treatment strategy and prevent the human and economic costs associated with delaying treatment. Combining molecular alterations at different levels (genomic, transcriptomic, and proteomic) can improve the accuracy of diagnosis, prognosis, and prediction of BC, and is a key prerequisite for successful individualized treatment strategies.

However, several key questions still need to be carefully addressed in the future to accelerate the adoption of biomarkers into clinical practice. (1) What biomarkers can help in the early detection of BC in high-risk populations? (2) What are the minimum requirements for biomarkers suitable to complement current routine screening for overall satisfactory accuracy? (3) Does liquid biopsy have the ability to differentiate between benign and malignant tumor? (4) What potential solutions can bridge the gap between basic research and clinical translation of these biomarkers?

### Future perspectives for precision treatment of BC

Over the past decade, precision medicine has evolved considerably. Liquid biopsy has revolutionized the field of clinical oncology considerably by facilitating tumor sampling, continuous monitoring through repeat sampling, the development of personalized treatment plans, and the screening for treatment resistance. Although liquid biopsy technology is still evolving, its noninvasive nature promises to revolutionize the precision treatment of BC. Additionally, we hypothesize that a shift from single to multiple markers could improve BC diagnosis, monitoring, and treatment. Active collaboration between clinicians, industrial scientists, and biologists is necessary.

Noninvasive liquid biopsy technology holds promise for clinical applications in the early diagnosis of BC, accurate drug administration, real-time documentation and monitoring of disease evolution, and prognostic assessment. At the same time, it is critical to conduct more multicenter, large-scale clinical trials to verify its clinical effectiveness.

## Data Availability

Not applicable.

## Abbreviations

BC: Bladder cancer
MIBC: Muscle-invasive bladder cancer
NMIBC: Non-muscle-invasive bladder cancer
CTCs: Circulating tumor cells
ICIs: Immune checkpoint inhibitors
cfRNA: Cell-free RNA
ctDNA: Circulating tumor deoxyribonucleic acid
miRNAs: MicroRNAs
lncRNAs: Long non-coding RNAs
circRNAs: Circular RNAs
EVs: Extracellular vesicles
EMT: Epithelial-mesenchymal transition
EpCAM: Epithelial cell adhesion molecule
cfDNA: Cell-free DNA
NGS: Next-generation sequencing
WGS: Whole genome sequencing
LC–MS: Liquid chromatography–mass spectrometry
GC–MS: Gas chromatography–mass spectrometry
AUC: Area under the receiver operating characteristics curve
OS: Overall survival
CSS: Cancer-specific survival
RFS: Recurrence-free survival
DFS: Disease-free survival
PFS: Progression free survival
TFR: Time to first recurrence
TSR: Time to second recurrence
TTP: Time to progression
NMR: Nuclear magnetic resonance
UCA1: Uroepithelial carcinoma-associated 1
UHPLC: Ultra-high performance liquid chromatography
NAC: Neoadjuvant chemotherapy
TUR: Transurethral resection of the urinary bladder
RC: Radical cystectomy
LNMAT2: Lymph node metastasis-associated transcript 2
CRK: CT10 regulator kinase
MRD: Minimal residual disease
PD-L1: Programmed death-ligand 1
